# Downregulation of cell division cycle-associated protein 7 (CDCA7) suppresses cell proliferation, arrests cell cycle of ovarian cancer, and restrains angiogenesis by modulating enhancer of zeste homolog 2 (EZH2) expression

**DOI:** 10.1080/21655979.2021.1965441

**Published:** 2021-09-23

**Authors:** Chunyan Cai, Xing Peng, Yumei Zhang

**Affiliations:** Department Of Gynaecology, The Affiliated Huai’an No.1 People’s Hospital of Nanjing Medical University, Huai’an, Jiangsu, China

**Keywords:** CDCA7, EZH2, ovarian cancer progression, angiogenesis

## Abstract

The purpose of the current study was to investigate the biological function of cell division cycle-associated protein 7 (CDCA7) on ovarian cancer (OC) progression and analyze the molecular mechanism of CDCA7 on OC cellular processes and angiogenesis. CDCA7 expression in OC tissues and adjacent normal tissues was obtained from Gene Expression Profiling Interactive Analysis (GEPIA) and in various cancer cell lines was obtained from Cancer Cell Line Encyclopedia (CCLE). Moreover, CDCA7 expression in adjacent normal tissues and tumor tissues of OC patients as well as in normal ovarian epithelial cells (NOEC) and ovarian cancer cells (OVCAR3, SKOV3, CAOV-3, A2780) was further confirmed via Western blot assay and Reverse transcription-quantitative polymerase chain reaction (RT-qPCR). In addition, Immunohistochemistry (IHC) was also applied for determination of CDCA7 expression in tissues of OC patients. Then, SKOV3 cells were introduced with shRNA-CDCA7 for functional experiments. GeneMANIA database analysis and coimmunoprecipitation (Co-IP) assay verified the interaction between CDCA7 and enhancer of zeste homolog 2 (EZH2) to probe the potential mechanism. CDCA7 expression was elevated in tumor tissues of OC patients and OC cell lines. CDCA7 silencing restrained the proliferative, migrative and invasive capacities and arrested cell cycle of OC cells. In addition, CDCA7 knockdown induced a weaker in vitro angiogenesis of HUVECs. Mechanistically, CDCA7 interacted with EZH2. Downregulation of CDCA7 arrested angiogenesis by suppressing EZH2 expression. To sum up, the current study revealed the impact and potential mechanism of CDCA7 on OC cellular processes, developing a promising molecular target for OC therapies.

## Graphical Abstract

**Figure:**
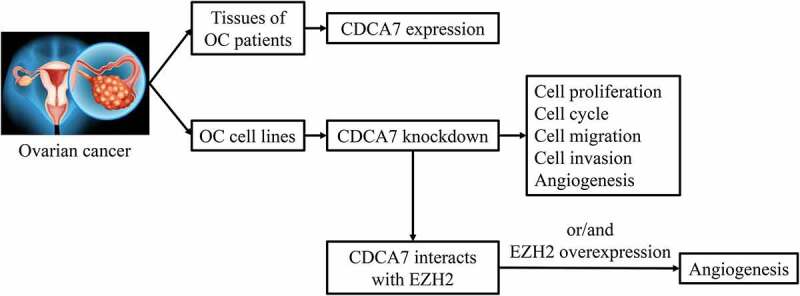

## Introduction

Cancer is the most serious disease that endangers human health and life at present, among which ovarian cancer (OC) is one of the three most common malignancies of female reproductive system [1]. OC, accompanied by atypical symptoms, is insidious at its early stage because the ovary is deeply located in the pelvic cavity. Most patients have suffered from terminal cancer at the time of diagnosis, which poses a serious threat to female reproductive health [2]. Although clinical remission could be achieved in some cases with cytoreductive surgery in combination with platinum-based chemotherapy, approximately 70% of advanced OC patients will relapse within 18–28 months [3]. Hence, there is an urgent need to develop novel insights into exploration of biomarkers for OC therapies.

Tumor is a class of cellular cyclical disease. A variety of cytokines participate in the regulation of cell cycle [4]. The cell division cycle-associated protein (CDCA) family are vital regulators of cell proliferation and cell cycle. CDCA7, located on chromosome 2q31, is first detected in Myc-transfected fibroblasts [5]. Numerous studies verify that CDCA7 is highly expressed in a variety of malignancies and closely associated with tumor progression, invasion and metastasis [6,7]. Elevated CDCA7 expression is linked to low survival rate and poor prognosis [8]. Importantly, Cho et al revealed that CDCA7 gene is upregulated in human OC cell lines [9]. However, interactions between CDCA7 and OC progression has not yet been elaborated.

The imbalance of epigenetic pathways often occurs in cancers. Understanding underlying mechanism of epigenetic factors helps to develop effective cancer therapies [10]. Polycomb repressive complex (PRC) is a model for the function of epigenetic regulatory factors in tumor cells [11]. Enhancer of zeste homolog 2 (EZH2), a histone-lysine N-methyltransferase enzyme encoded by EZH2 gene, is a key regulator of PRC [12]. Moreover, EZH2 is responsible for trimethylation of histone H3 on Lys27 (H3K27me3) that results in epigenetic gene silencing. Recent research indicates that EZH2 is overexpressed in various tumor tissues and closely related to tumor infiltration, metastasis and prognosis [13,14]. Accordingly, inhibition of EZH2 might be a promising strategy for therapies of cancers in the future.

Here, this current work was carried out to identify the biological role of CDCA7 in OC. The impact of CDCA7 on OC cellular processes was analyzed through a series of functional experiments. What’s more, potential molecular mechanism of CDCA7 on angiogenesis in OC was investigated in vitro.

## Materials and methods

### In silico analysis

Expression levels of CDCA7 in OC tissues and normal tissues were obtained at Gene Expression Profiling Interactive Analysis (GEPIA; http://gepia.cancer-pku.cn/index.html). Cancer Cell Line Encyclopedia (CCLE; https://portals.broadinstitute.org/ccle/) database was applied to interrogate CDCA7 expression in OC cell lines.

### Clinical sample collection

A total of five pairs of clinical tumor tissue specimens and adjacent normal ones were collected from OC patients. This study was approved by the Ethics Committee of the Affiliated Huaian No. 1 People’s Hospital of Nanjing Medical University. Patients and their families agreed to participate in the study and signed the informed consent.

### Cell culture

Normal ovarian epithelial cells (NOEC), ovarian cancer cells (OVCAR3, SKOV3, CAOV-3, A2780), and human umbilical vein endothelial cells (HUVECs) were obtained from the Institute of Biochemistry and Cell Biology, Chinese Academy of Sciences (Shanghai, China). Cells were cultured in Dulbecco’s modified Eagle’s medium (DMEM; Invitrogen, CA, USA) containing 10% fetal bovine serum (FBS; Invitrogen, CA, USA), penicillin (100 U/mL)/streptomycin (100 μg/mL) (Invitrogen, CA, USA) in a humidified chamber at 37°C with 5% CO_2_.

### Cell transfection

The lentivirus (GenePharma, Shanghai, China) carrying EZH2 gene (OV-EZH2), shRNA-CDCA7, or negative control (OV-NC or shRNA-NC) were transfected with Lipofectamine 2000 transfection reagent (Invitrogen, CA, USA) following the manufacturer’s instructions. Briefly, cells (2×x10^5/^well) were seeded into 24-well plates. About 4 µg vectors were mixed with 20 µl Lipofectamine 2000 in Opti-MEM (Invitrogen, CA, USA) at room temperature for 15 min. Then, the mixture was added into the plate and transfections were performed at room temperature for 6 h. Subsequently, the medium was replaced with fresh medium and transfected cells were then cultured for 48 h before the subsequent experiments.

### Immunohistochemistry (IHC)

Briefly, tumor tissues were fixed and cut into 4-μm-thick sections. Then, sections were deparaffinized in xylene and dehydrated in a graded ethanol series. Next, sections were immersed in sodium citrate and heated for antigen retrieval. After incubation with 3% H_2_O_2_ methanol solution for 15 min, sections were blocked with 10% goat serum and then incubated overnight at 4°C with anti-CDCA7 (Sigma-Aldrich, HPA005565, 1:50). The secondary antibody was used to incubate the sections for 20 min at room temperature. Diaminobenzidine (DAB; Solarbio, Beijing, China) was applied for staining development and sections were counterstained with hematoxylin for 10 min. The staining results were observed under a light microscope (Leica, Wetzlar, Germany).

### Counting kit-8 (CCK-8) assay

SKOV3 cells were collected and seeded into 96-well plates at a density of 10^4^ cells/well. Cells were cultured for 24, 48, and 72 h and then 10 μL CCK-8 reagent (Beyotime, Shanghai, China) were added into each well. Next, cells were continuously cultured for 2 h. Finally, OD450 (optical density at 450 nm) values were detected by a microplate reader (BioTek, Vermont, USA).

### Flow cytometry

In brief, SKOV3 cells were digested with trypsin and washed with PBS. Then, cells were fixed with 70% ice-cold ethanol at 4°C for 4 h. Afterward, cells were stained with propidium iodide (PI; Beyotime, Shanghai, China) at room temperature in the dark for 30 min and assessed by a FACS flow cytometer (BD Biosciences, CA, USA).

### Wound healing assay

The migrative ability of OC cells was evaluated by wound healing assay. Briefly, SKOV3 cells were spread onto 6-well plates. When reaching 90% confluence, SKOV3 cells were divided with a 200 μl of pipette tip into a cell-free area. Next, serum-free medium was added for culture. After 24 h incubation, the migration distance was imaged and evaluated under an inverted microscope (Leica, Wetzlar, Germany).

### Transwell invasion assay

Transwell chambers precoated with Matrigel (BD Biosciences, CA, USA) were placed into 24-well plates. Serum-free culture solution containing 1 × 10^5^ cells was appended to the upper chamber, and 500 μL complete medium was added to the lower chamber. Post 24-h incubation, transwell chambers were washed with PBS and fixed by 4% paraformaldehyde for 10 min. The invaded cells were stained with 0.1% crystal violet (Solarbio, Beijing, China) and observed under an inverted microscope (Leica, Wetzlar, Germany).

### Tube formation assay

96-well plates were pre-coated with 100 µl Matrigel. HUVECs were resuspended with serum-free medium at a density of 2 × 10^5^/ml. Then, 100 µl of HUVECs was seeded on the Matrigel and cultured for 6 h. Tube formation was observed and photographed using a light microscope (Leica, Wetzlar, Germany). The number of tube-like structures was determined using Image J software (National Institutes of Health, MD, USA).

### Reverse transcription-quantitative polymerase chain reaction (RT-qPCR)

TRIzol Reagent (Invitrogen, CA, USA) was used to extract total RNA from OC tissues and cells. Then, 5 μg of total RNA was used to prepare complementary deoxyribose nucleic acid (cDNA) by PrimeScript™ RT Reagent Kit (TaKaRa, Tokyo, Japan). Real-time quantitative PCR was performed using ABI 7500 quantitative PCR instrument (ABI/Perkin Elmer, CA, USA). The PCR thermocycling conditions were as follows: 10 min at 95°C, followed by 40 cycles of 95°C for 15 sec and 60°C for 30 sec. The following primer sequences were used for qPCR: CDCA7: forward, 5ʹ-CCAGGCTCCGACTCACAATCAAG-3ʹ and reverse, 5ʹ-GTACTTATCCTCTTCCTCCTCCTCCTC-3ʹ; EZH2: forward, 5ʹ-AATCAGAGTACATGCGACTGAGA-3ʹ and reverse, 5ʹ-GCTGTATCCTTCGCTGTTTCC-3ʹ; GAPDH: forward, 5ʹ-TGACTTCAACAGCGACACCCA-3ʹ and reverse, 5ʹ-CACCCTGTTGCTGTAGCCAAA-3ʹ. GAPDH served as the internal reference. Relative gene expression of CDCA7 and EZH2 was analyzed by 2^−ΔΔCt^ method.

### Western blot assay

Total protein from OC tissues and cells was extracted using protein extraction lysate (Beyotime, Shanghai, China). Then, protein concentration was evaluated by BCA Protein Assay Kit (Beyotime, Shanghai, China). Protein samples were separated by 10% SDS-PAGE and subsequently transferred to polyvinylidene difluoride (PVDF) membranes (Millipore, MA, USA). Next, PVDF membranes were blocked with 5% nonfat milk for 2 h and incubated overnight at 4°C with primary antibody against CDCA7 (Thermo Fisher Scientific, PA5-101,299, 1:1000), EZH2 (Thermo Fisher Scientific, MA5-15,101, 1:1000), CyclinE1 (Abcam, ab33911, 1:1000), CyclinE2 (Abcam, ab40890, 1:5000), MMP2 (Abcam, ab97779, 1:2000), MMP9 (Abcam, ab228402, 1:1000), VEGFA (Abcam, ab214424, 1:1000), VEGFR1 (Abcam, ab32152, 1:5000), VEGFR2 (Abcam, ab134191, 1:5000) and GAPDH (Thermo Fisher Scientific, MA5-15,738-D680, 1:1000). The next day, membranes were washed with TBST and incubated with the corresponding secondary antibody (Thermo Fisher Scientific, A32728, 1:10,000) for 2 h at room temperature. Enhanced chemiluminescence reagents (ECL; Pierce, IL, USA) were applied to visualize the protein bars. Finally, protein bands were detected by a Bio-Rad imaging system (Bio-Rad, CA, USA).

### GeneMANIA database analysis

GeneMANIA (http://genemania.org/) was an analytical tool to analyze the interconnection of gene clusters. We constructed gene interaction networks for CDCA7 using GeneMANIA.

### Coimmunoprecipitation (Co-IP)

Cells were lysed by RIPA buffer containing protease inhibitors (Beyotime, Shanghai, China). The supernatant was incubated overnight at 4°C with anti-CDCA7, anti-EZH2 antibody or IgG as negative control. Then, 20 μL protein A/G-beads was coincubated with the supernatant at 4°C for 1 h. The beads were extracted and washed three times with lysis buffer. The immunocomplexes were used for western blotting assay.

### Data analysis

All data analyses were processed with SPSS 21.0 statistical software (IBM, NY, USA) and measurement data from three independent experiments were expressed as means ± standard deviation (SD). One-way analysis of variance (ANOVA) followed by Tukey’s post hoc test was used for comparisons among multiple groups. The p value < 0.05 indicate a statistically significant difference.

## Results

### Elevated CDCA7 expression in tumor tissues of OC patients

**Figure 1.**
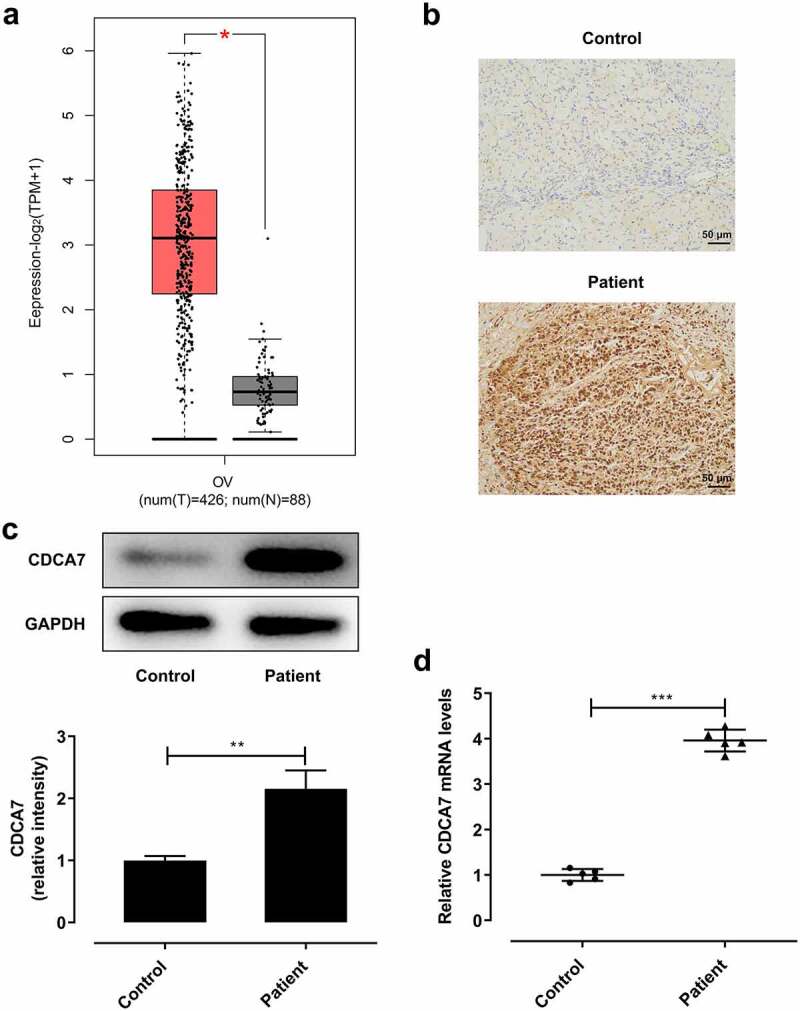
Elevated CDCA7 expression in tumor tissues of OC patients

Quantification analysis of CDCA7 levels in tumor tissues and adjacent normal tissues of OC patients was done in GEPIA database. A relatively higher level of CDCA7 was observed in tumor tissues of OC patients (Figure 1(a)). Furthermore, CDCA7 levels in five pairs of clinical tumor tissue specimens and adjacent normal ones were assessed by performing IHC, western blot and RT-qPCR assay. IHC staining confirmed the high expression of CDCA7 in tumor tissues compared to the adjacent non-tumor tissues (Figure 1(b)). Consistently, in comparison with adjacent normal tissues, CDCA7 protein (Figure 1(c)) and mRNA (Figure 1(d)) levels were significantly upregulated in tumor tissues of OC patients.

(a) Expression levels of CDCA7 in OC tissues and adjacent normal tissues were obtained at Gene Expression Profiling Interactive Analysis (GEPIA; http://gepia.cancer-pku.cn/index.html). (b) IHC staining for determination of CDCA7 expression in 5 pairs of clinical tumor tissue specimens and adjacent normal ones. (c) Western blot assay for determination of CDCA7 protein level in 5 pairs of clinical tumor tissue specimens and adjacent normal ones. (d) RT-qPCR for determination of CDCA7 mRNA level in five pairs of clinical tumor tissue specimens and adjacent normal ones. *p < 0.05, **p < 0.01, ***p < 0.001.

### Elevated CDCA7 expression in OC cell lines

**Figure 2.**
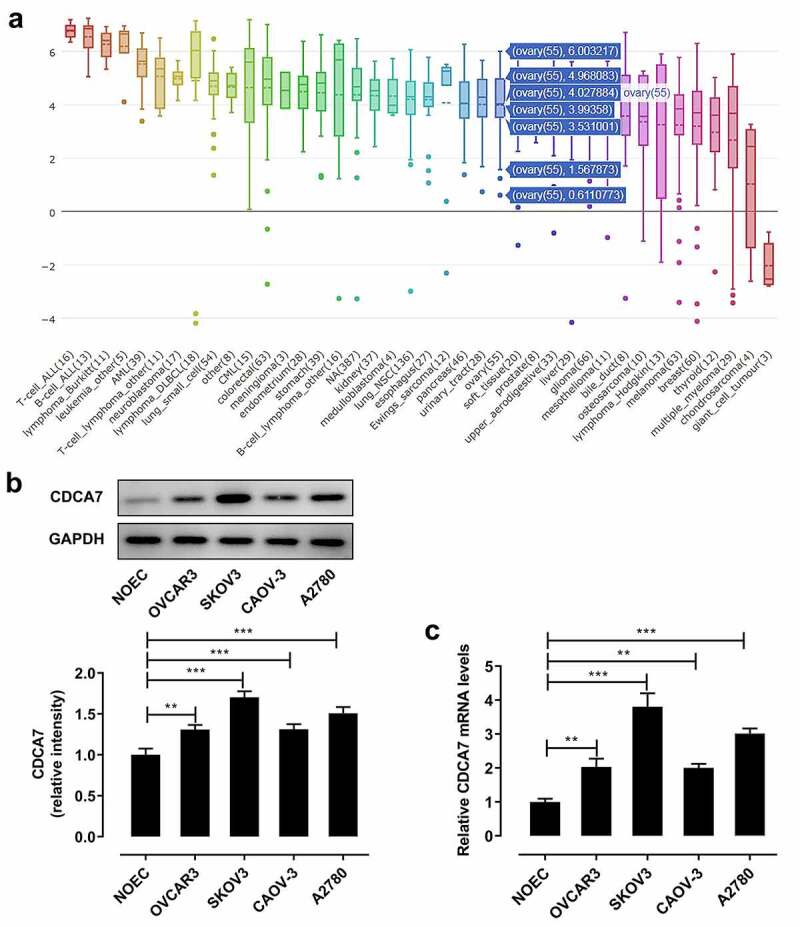
Elevated CDCA7 expression in OC cell lines

CDCA7 expression in various cancer cell lines were assessed in CCLE database. Importantly, a relatively higher level of CDCA7 was seen in OC cell lines (Figure 2(a)). Moreover, differences of CDCA7 expression in normal ovarian epithelial cells (NOEC) and ovarian cancer cells (OVCAR3, SKOV3, CAOV-3, A2780) were assessed. Compared with that in NOEC cells, CDCA7 protein (Figure 2(b)) and mRNA (Figure 2(c)) levels in ovarian cancer cells (OVCAR3, SKOV3, CAOV-3, A2780) were distinctly upregulated, especially in SKOV3 cells. Hence, SKOV3 cells were chosen for the subsequent experiments.

(a) Expression levels of CDCA7 in various cancer cell lines were obtained at Cancer Cell Line Encyclopedia (CCLE; https://portals.broadinstitute.org/ccle/) database. (b) Western blot assay for determination of CDCA7 protein level in normal ovarian epithelial cells (NOEC) and ovarian cancer cells (OVCAR3, SKOV3, CAOV-3, A2780). (c) RT-qPCR for determination of CDCA7 mRNA level in normal ovarian epithelial cells (NOEC) and ovarian cancer cells (OVCAR3, SKOV3, CAOV-3, A2780). **p < 0.01, ***p < 0.001.

### CDCA7 silencing suppressed OC cell proliferation and arrested cell cycle

**Figure 3.**
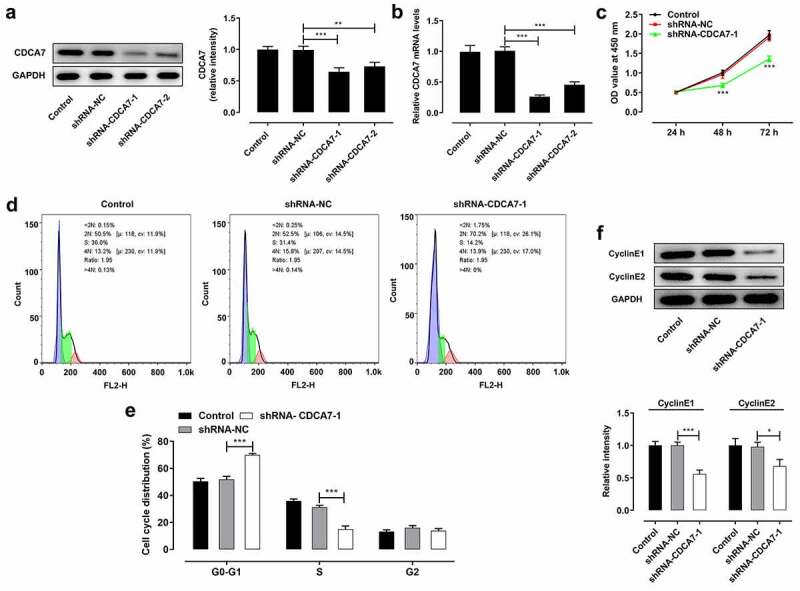
CDCA7 silencing arrested OC progression

To investigate the biological functions of CDCA7 on OC progression, shRNA-CDCA7-1 or shRNA-CDCA7-2 were introduced into SKOV3 cells. About 48-h posttransfection, CDCA7 protein (Figure 3(a)) and mRNA (Figure 3(b)) levels were markedly downregulated in SKOV3 cells. Due to the optimized transfection efficiency, shRNA-CDCA7-1 was chosen for the functional experiments. CDCA7 silencing prominently restrained OC cell proliferation (Figure 3(c)). Additionally, flow cytometry analysis of cell cycle distribution revealed that CDCA7 knockdown visibly induced cell cycle arrest of OC cells (Figure 3(d,e)). What’s more, decreases of CyclinE1 and CyclinE2 protein levels further demonstrated that CDCA7 silencing can arrest cell cycle in OC (Figure 3(f)).

(a) Western blot assay was applied to validate the transfection efficiency in SKOV3 cells following introduction of shRNA-CDCA7-1 or shRNA-CDCA7-2. (b) RT-qPCR was applied to validate the transfection efficiency in SKOV3 cells following introduction of shRNA-CDCA7-1 or shRNA-CDCA7-2. (c) CCK-8 assay for determination of OC cell proliferation. (d) Flow cytometry analysis for determination of OC cell cycle. (e) Quantitative analysis of cell cycle distribution of SKOV3 cells. (f) Western blot assay for determination of CyclinE1 and CyclinE2 protein levels. *p < 0.05, **p < 0.01, ***p < 0.001.

### CDCA7 silencing repressed OC cell migration and invasion capacities

**Figure 4.**
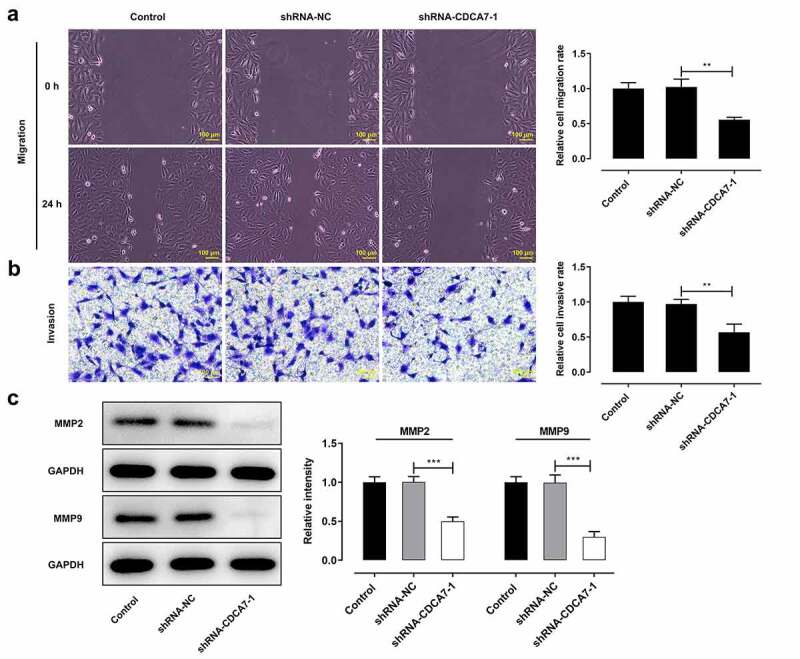
CDCA7 silencing restrained the migrative and invasive abilities of OC cells

It was observed that CDCA7 silencing strongly restrained the migration of SKOV3 cells. In addition, the invasive abilities of SKOV3 cells showed the similar tendency to be suppressed by CDCA7 knockdown (Figure 4(a,b)). Moreover, visible decreases of MMP2 and MMP9 further confirmed the suppressing effects of CDCA7 silencing on OC cell migration and invasion in vitro (Figure 4(c)).

(a) Wound healing assay for determination of the migration of SKOV3 cells. (b) Transwell assay for determination of the invasion of SKOV3 cells. (c) Western blot assay for determination of MMP2 and MMP9 protein levels. **p < 0.01, ***p < 0.001.

### CDCA7 silencing induced a weaker in vitro angiogenesis

**Figure 5.**
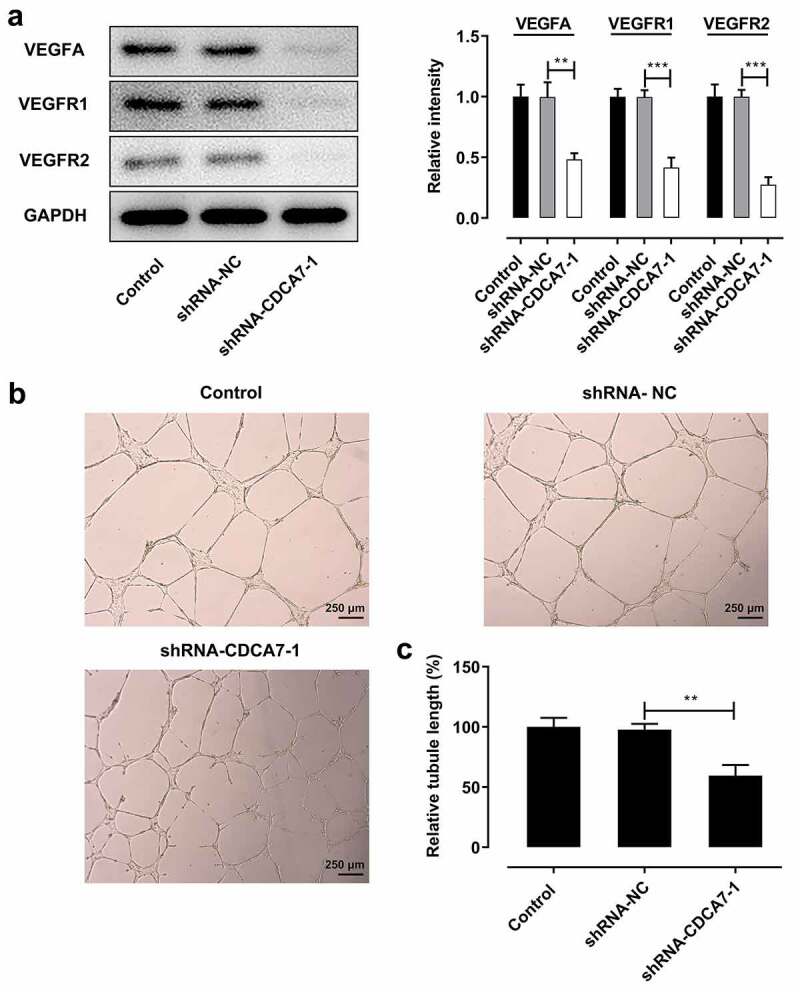
CDCA7 silencing repressed in vitro angiogenesis of HUVECs

It is well known that tumor growth and metastasis need glorious angiogenesis for nutrition provision. Obvious reduced VEGFA, VEGFR1, and VEGFR2 expression indicated that CDCA7 silencing arrested angiogenesis (Figure 5(a)). Moreover, tube formation assay of HUVECs also revealed that angiogenesis was suppressed by downregulation of CDCA7 (Figure 5(b,c)). Together, these results suggested that CDCA7 knockdown induced a weaker in vitro angiogenesis of HUVECs.

a) Western blot assay for determination of VEGFA, VEGFR1, and VEGFR2 protein levels. (b) Tube formation assay of HUVECs. (c) Quantitative analysis of the angiogenesis ability. **p < 0.01, ***p < 0.001.

### CDCA7 interacted with EZH2

**Figure 6.**
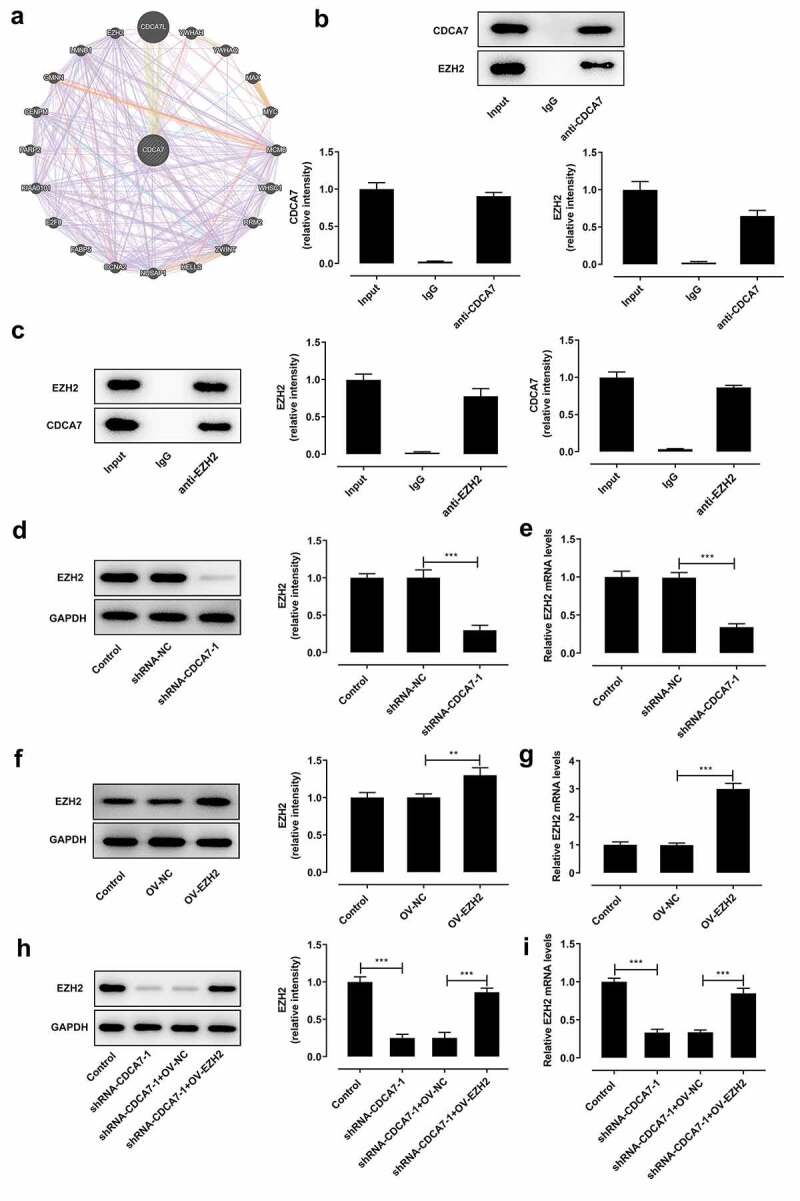
CDCA7 interacted with EZH2

GeneMANIA database analysis presented that CDCA7 interacted with EZH2 (Figure 6(a)). Here, we further verified the interaction between CDCA7 and EZH2 through Co-IP assay. EZH2 existed in anti-CDCA7 group (Figure 6(b)) and CDCA7 existed in anti-EZH2 group (Figure 6(c)). EZH2 protein (Figure 6(d)) and mRNA (Figure 6(e)) levels were restrained by CDCA7 silencing, which prompted a positive correlation between CDCA7 and EZH2 expression. EZH2 protein (Figure 6(f)) and mRNA (Figure 6(g)) levels were markedly elevated following transfection with EZH2 overexpression plasmid. Furthermore, introduction of EZH2 overexpression plasmid elevated EZH2 protein (Figure 6(h)) and mRNA (Figure 6(i)) levels, partly abolishing the suppressing effects of CDCA7 silencing on EZH2 expression.

(a) GeneMANIA database analysis of the interaction between CDCA7 and EZH2. (b, c) Co-IP assay of the interaction between CDCA7 and EZH2. (d, e) SKOV3 cells were introduced with shRNA-CDCA7. Western blot assay and RT-qPCR were applied to assess the regulating effects of CDCA7 knockdown on EZH2 protein and mRNA levels. (f, g) SKOV3 cells were introduced with EZH2 overexpression plasmid. Western blot assay and RT-qPCR were applied to evaluate the transfection efficiency. (h, i) SKOV3 cells were transfected with shRNA-CDCA7 or cotransfected with shRNA-CDCA7 and EZH2 overexpression plasmid. Western blot assay and RT-qPCR were applied to assess the influence of EZH2 overexpression plasmid on the regulating effects of CDCA7 knockdown. **p < 0.01, ***p < 0.001.

### Downregulation of CDCA7 arrested angiogenesis by suppressing EZH2 expression

**Figure 7.**
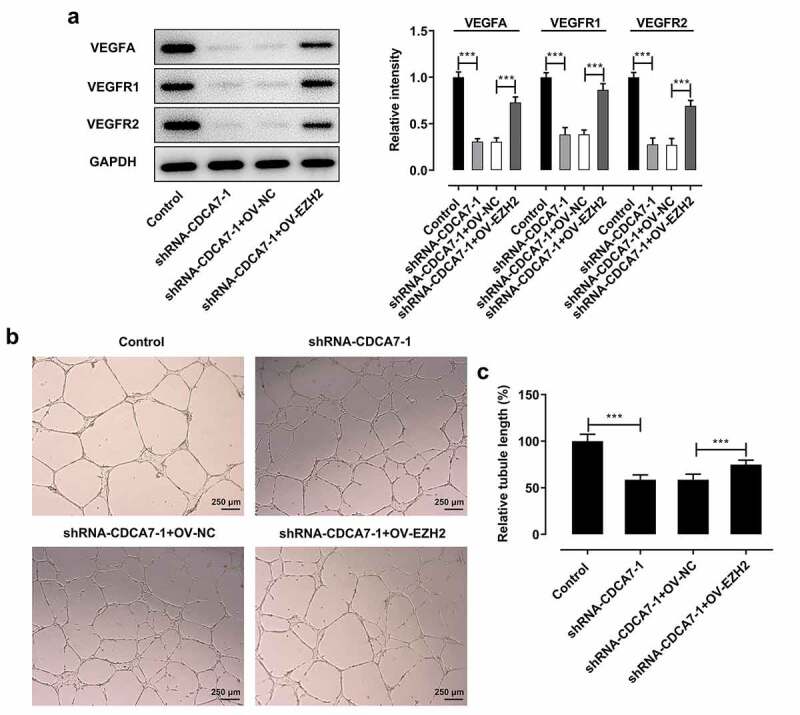
CDCA7 silencing arrested angiogenesis by suppressing EZH2 expression

It was seen that suppressing effects of CDCA7 silencing on VEGFA, VEGFR1, and VEGFR2 expression were abolished upon EZH2 elevation (Figure 7(a)). What’s more, tube formation assay of HUVECs together confirmed that the weaker angiogenesis induced by CDCA7 knockdown was partly reversed following EZH2 overexpression (Figure 7(b,c)).

(a) Western blot assay for determination of VEGFA, VEGFR1, and VEGFR2 protein levels. (b) Tube formation assay of HUVECs. (c) Quantitative analysis of the angiogenesis ability. ***p < 0.001.

## Discussion

In recent years, OC treatment mainly focuses on targeted therapy and surgery. Accompanied by in-depth research on the pathogenesis of OC, great progress has been made in OC therapies [15]. However, despite continuous improvement in therapy, the overall 5-year survival rate of OC patients at advanced stages was less than 50% [16].

Multiple genes and proteins are involved in the process of cell cycle regulation, and CDCA7 is one of them. More and more studies have found that abnormal expression of CDCA7 plays an important role in the occurrence and development of tumors [6,7]. In addition, it has been proved that CDCA7 gene is highly expressed in human OC cell line [9]. In this study, we noticed that CDCA7 was remarkably elevated in the collected tumor tissues of OC patients and OC cell lines compared to the normal ones. Additionally, downregulation of CDCA7 suppressed OC cell growth and arrested at G0/G1 phase of the cell cycle.

Tumor metastasis is the main cause leading to poor prognosis of cancer patients. The influence of CDCA7 on tumor metastasis has gradually attracted the attention of researchers [8]. A growing number of studies have identified that matrix metalloproteinases (MMPs) play crucial roles in the multistep process of tumor metastasis. MMP-2 and MMP-9 belong to the gelatinases in MMPs. They can develop into type IV collagenase, then degrade extracellular matrix and destroy the complete basement membrane, allowing cancer cells to infiltrate the surrounding tissue and invade blood and lymphatic vessels [17,18]. Our current work demonstrated that knockdown of CDCA7 inhibited OC cell migration and invasion and suppressed MMP-2 and MMP-9 expression. What’s more, tumor growth and metastasis depend on angiogenesis. Vascular endothelial growth factor (VEGF) signaling pathway plays a key role in tumor-associated angiogenesis [19]. As a matter of fact, a lot of anti-tumor angiogenesis drugs targeting VEGF/VEGFR have recently entered in clinical application [20,21]. In the present study, it was observed that CDCA7 depletion repressed the expressions of VEGFA, VEGFR1 and VEGFR2 in OC cells and blood vessels formation of vascular endothelial cells.

As an important component of polycomb group proteins (PcGs), EZH2 has been confirmed to be overexpressed in a variety of cancer types with lethal outcomes, including OC cases [13,14]. The EZH2 gene mainly contributes to tumor development by promoting angiogenesis, silencing tumor suppressor genes and inhibiting the apoptosis of tumor cells [22,23]. In addition, it has been widely demonstrated that EZH2 is closely associated with OC progression, invasion and metastasis [24,25]. More importantly, GeneMANIA database analysis shows that CDCA7 can interact with EZH2. Here, in the present work, it was observed that knockdown of CDCA7 could suppress EZH2 expression, indicating a positive regulation between CDCA7 and EZH2. As expected, overexpression of EZH2 could reverse the inhibitory effects of CDCA7 interference on OC angiogenesis.

## Conclusion

To conclude, the current study validated that CDCA7 was highly expressed in OC tissues and cell lines. Furthermore, functional experiments evidenced that knockdown of CDCA7 exhibited inhibitory effects on OC cell growth, migration, invasion, and angiogenesis in vitro. Mechanically, downregulation of CDCA7 arrested angiogenesis by suppressing EZH2 expression. These findings prompted that CDCA7 may be a promising molecular target for OC therapies.

## Data Availability

The datasets used and/or analyzed during the present study are available from the corresponding author on reasonable request.
